# Activation of TGF-β1-CD147 positive feedback loop in hepatic stellate cells promotes liver fibrosis

**DOI:** 10.1038/srep16552

**Published:** 2015-11-12

**Authors:** Hai-Yan Li, Di Ju, Da-Wei Zhang, Hao Li, Ling-Min Kong, Yanhai Guo, Can Li, Xi-Long Wang, Zhi-Nan Chen, Huijie Bian

**Affiliations:** 1 State Key Laboratory of Cancer Biology, Cell Engineering Research Center and Department of Cell Biology, Fourth Military Medical University, Xi′an 710032, China; 2 Department of Medical Technology, Xi′an Medical University, Xi′an, 710021 China; 3 Research Center for Biological Therapy, Beijing 302 Hospital, Beijing, China; 4 Department of Pharmacogenomics, School of Pharmacy, Fourth of Military Medical University, Xi′an, 710021 China

## Abstract

Activation of hepatic stellate cells (HSCs) by transforming growth factor-β1 (TGF-β1) initiates HBV-associated fibrogenesis. The mechanism of TGF-β1 modulating HSC activation is not fully uncovered. We hypothesized a positive feedback signaling loop of TGF-β1-CD147 promoting liver fibrogenesis by activation of HSCs. Human HSC cell line LX-2 and spontaneous liver fibrosis model derived from HBV transgenic mice were used to evaluate the activation of molecules in the signaling loop. Wound healing and cell contraction assay were performed to detect the CD147-overexpressed HSC migration and contraction. The transcriptional regulation of CD147 by TGF-β1/Smad4 was determined using dual-luciferase reporter assay and chromatin immunoprecipitation. We found that a positive reciprocal regulation between TGF-β1 and CD147 mediated HSC activation. CD147 over-expression promoted HSC migration and accelerated TGF-β1-induced cell contraction. Phosphorylation of Smad2 and Smad3 in cooperation with Smad4 mediated the TGF-β1-regulated CD147 expression. Smad4 activated the transcription by direct interaction with CD147 promoter. Meanwhile, CD147 modulated the activated phenotype of HSCs through the ERK1/2 and Sp1 which up-regulated α-SMA, collagen I, and TGF-β1 synthesis. These findings indicate that TGF-β1-CD147 loop plays a key role in regulating the HSC activation and combination of TGF-β receptor inhibitor and anti-CD147 antibody might be promised to reverse fibrogenesis.

Liver fibrosis results from chronic liver injury during a long-term wound-healing response, which causes increasing excessive accumulation of extracellular matrix (ECM) proteins and eventually leads to fibrogenesis and later cirrhosis^1^. The hepatic stellate cells (HSCs) are the main ECM-producing cells during this process, and they activate and differentiate from quiescent vitamin A-storing cells into proliferative myofibroblasts in response to fibrogenic liver injury. Activated HSCs express many ECM proteins including collagen type I, α-smooth muscle actin (α-SMA), transforming growth factor-β1 (TGF-β1), matrix metalloproteinase (MMP), and tissue inhibitors of metalloproteinases, which contributes to liver fibrosis^2^. Clinical studies suggest that hepatitis B virus (HBV) chronic infection is the most important cause of liver cirrhosis and hepatocellular carcinoma (HCC) in human patients^3^. TGF-β1 is considered a key mediator of liver fibrogenesis and detected in HBV-related liver fibrogenesis^4^,^5^. The TGF-β1 gene is transcriptionally activated by hepatitis B virus X protein (HBx) which is one of HBV encoded-proteins through the Egr-1 binding sites^6^.

Liver-damage-induced levels of active TGF-β1 mediate HSC transdifferentiation through the canonical Smad signaling pathway involving TGF-β receptor-mediated phosphorylation of Smad2 and Smad3 (p-Smad2/3) to enhance collagen synthesis^7^. The p-Smad2/3 form complexes with Smad4, which are translocated to the nucleus to regulate the transcription of certain genes. Putative target genes of Smad4 are screened by promoter-wide analysis in human epithelial cells^8^. However, the target genes transcriptionally regulated by Smad4 in HSCs are unknown.

Our previous study and others′ reveal that a glycosylated transmembrane protein, CD147 presents on HSCs^9^,^10^. CD147 expression in HSCs is elevated by TGF-β1 stimulation^9^, but the regulating mechanism is not uncovered. In this study, we hypothesized a direct role of TGF-β1 in the development of liver fibrosis by the activation of HSCs through TGF-β1-CD147 signaling loop. We here showed that TGF-β1 was released from hepatocytes which was transfected by HBx, and exerted on HSC activation by directly transcriptional regulation of CD147 through TGF-β1/Smad4 signaling pathway. Over-expression of CD147 was positively feedback on TGF-β1 expression via the ERK1/2/Sp1 transduction. The TGF-β1-CD147 loop contributed to HBV-associated liver fibrosis progression.

## Results

### A positive reciprocal regulation between TGF-β1 and CD147 in HSC activation

**Figure 1:**
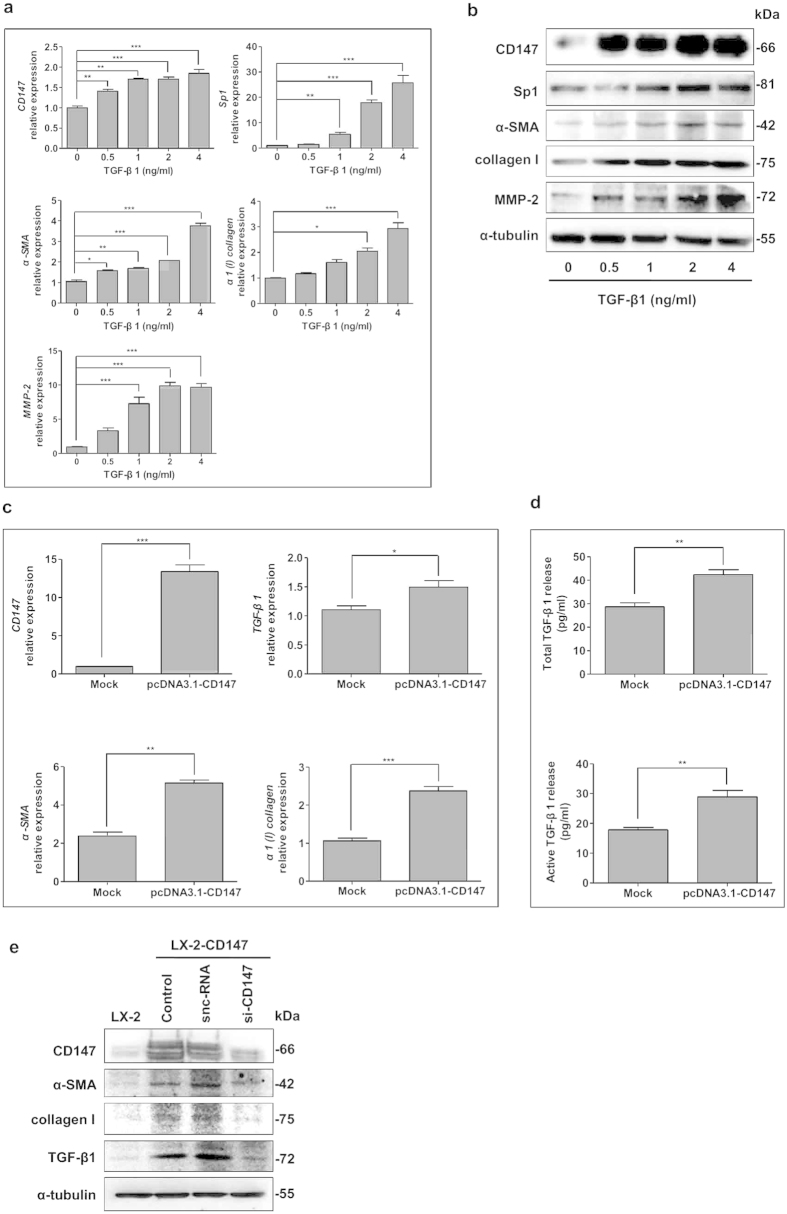
A positive reciprocal regulation between TGF-β1 and CD147 in HSC activation. (**a**) Real-time RT-PCR and (**b**) western blot analysis of CD147, Sp1, α-SMA, collagen I, and MMP-2 stimulated with various doses of TGF-β1 for 24 hours. (**c**) Real-time RT-PCR analysis of CD147, TGF-β1, α-SMA, and α1(I) collagen in LX-2 cells transiently transfected with plasmid pcDNA3.1-CD147 at 24 hours. (**d**) ELISA detection of total and active forms of TGF-β1 in supernatant of LX-2-transfected pcDNA3.1-CD147 cells. Cells were transfected with pcDNA3.1(+) as a mock control. (**e**) Western blot analysis of CD147, α-SMA, collagen I, and TGF-β1 in LX-2-CD147 cells treated with siRNA targeting CD147 (si-CD147). snc-RNA was used as a control. *P < 0.05, **P < 0.01, ***P < 0.001.

It is found that HSCs exposed to conditioned medium from HBx-expressing hepatocytes show increased expression of TGF-β1^11^,^12^. We confirmed that the ectopic expression of HBx in L02 cells (named L02-HBx) significantly induced the elevation of total and active TGF-β1 levels compared with controls (Supplemental Fig. 1a,b). Strikingly, we observed that CD147 was significantly increased in LX-2 cells either incubation with L02-HBx conditioned medium or co-cultured with L02-HBx cells. This up-regulation was inhibited with a selective antagonist of TGF-β1 type I receptor SB431542 (Sigma, St Louis, MO, USA), which demonstrated that TGF-β1 signaling transduction was involved in CD147 expression by a paracrine way (Supplemental Fig. 1c). We then evaluated the levels of CD147 and fibrosis-related genes in response to different doses of TGF-β1 in LX-2 cells. The mRNA and protein levels of CD147, α-smooth muscle actin (α-SMA), α1(I) collagen, and MMP-2 were significantly up-regulated with TGF-β1 stimulation in dose-dependent manners. A transcription factor Sp1 was also markedly increased by TGF-β1 (Fig. 1a,b). Meanwhile, Real-time RT-PCR analysis showed that the transfection of CD147 gene in LX-2 cells induced the increased mRNA expressions of TGF-β1, α-SMA, and α1(I) collagen (Fig. 1c). Also, both total and active forms of TGF-β1 were up-regulated by CD147 over-expression as detected by enzyme-linked immunosorbent assay (ELISA) (Fig. 1d). As endogenous level of CD147 was very low in quiescent LX-2 cells, thus we generated a LX-2-CD147 cell line that stably expressed CD147 by puromycin selection. Then we knocked down CD147 by treating LX-2-CD147 cells with a small interference RNA specific to CD147 (si-CD147). As shown in Fig. 1e, the elevated expression of CD147 in LX-2-CD147 cells was depressed by si-CD147 efficiently, which was coupled with down-regulation of α-SMA, collagen I, and TGF-β1. Collectively, our data suggested that HBx was capable of inducing the secretion of TGF-β1 in hepatocytes that resulted in paracrine activation of HSCs by CD147-TGF-β1 positive feedback loop transduction.

### Over-expression of CD147 promoted HSC transdifferentiation

**Figure 2:**
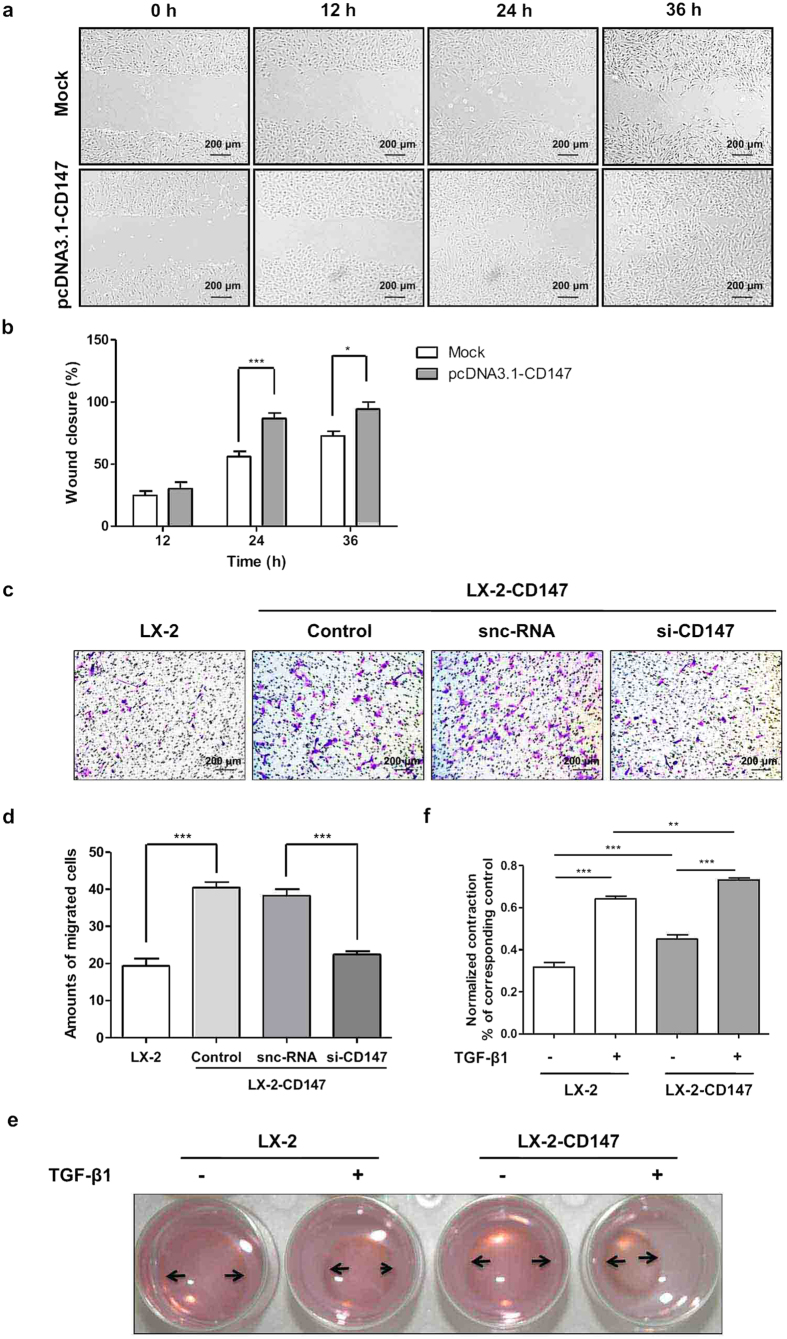
Over-expression of CD147 accelerated HSC migration and contraction. (**a**) Representative phase contrast image and (**b**) quantitative analysis of wound healing assay in LX-2-transfected pcDNA3.1-CD147 cells and mock controls. (**c**) Representative phase contrast image and (**d**) quantitative analysis of transwell migration assay in LX-2 and LX-2-CD147 cells which were treated with siRNA targeting CD147 (si-CD147). snc-RNA was used as a control. (**e**) Representative phase contrast image and (**f**) quantitative analysis of collagen-based cell contraction in LX-2-CD147 and LX-2 cells with or without TGF-β1 stimulation for 24 hours. *P < 0.05, **P < 0.01, ***P < 0.001.

As shown in Fig. 2a,b, transient over-expression of CD147 promoted LX-2 cell migration as detected by wound healing assay. Moreover, the transwell migration assay confirmed that the migration of LX-2 cells was accelerated by CD147 up-regulation, whereas, knockdown of CD147 by si-CD147 significantly decreased the migration of LX-2-CD147 cells compared with that of silencer-negative control siRNA (snc-RNA, Fig. 2c,d). With LX-2-CD147 cell model, collagen-based cell contraction assay showed that CD147 promoted HSC contraction and even accelerated TGF-β1-induced cell contraction (Fig. 2e,f). These results indicated that CD147 cooperated with TGF-β1 in HSC transdifferentiation.

### Smads signaling was involved in TGF-β1-induced CD147 expression

**Figure 3:**
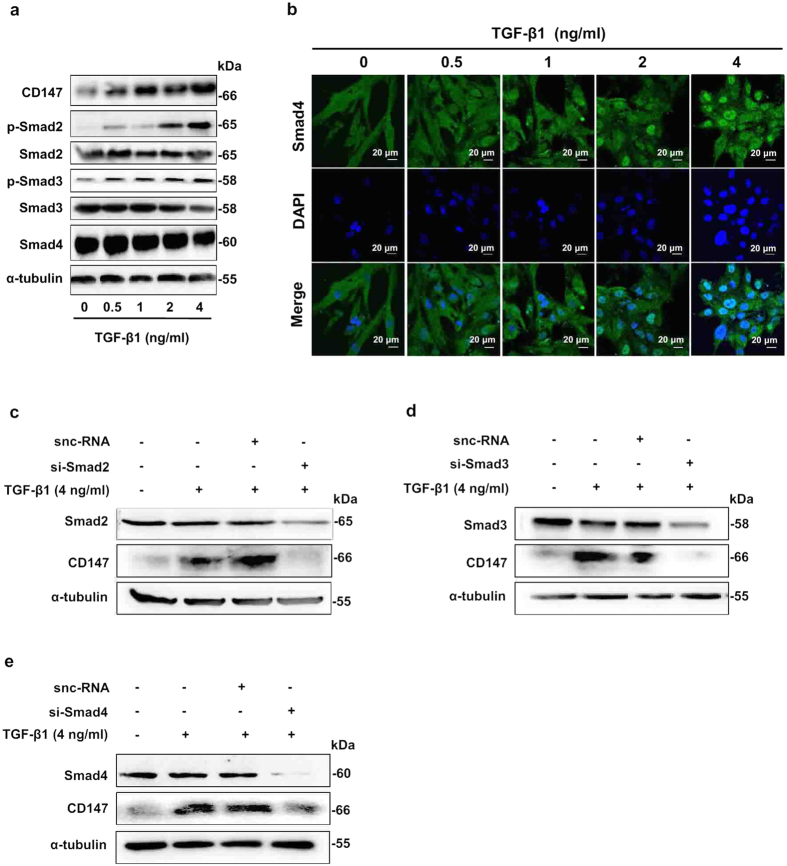
Smads signaling was involved in TGF-β1-induced CD147 expression. (**a**) Western blot analysis of CD147, p-Smad2, Smad2, p-Smad3, Smad3, and Smad4 expressions in LX-2 cells stimulated with various doses of TGF-β1 for 24 hours. (**b**) Immunofluorescent staining of Smad4 in LX-2 cells stimulated with TGF-β1. Cell nuclei were stained with DAPI. Western blot analysis of CD147 expression in LX-2 cells which were transfected with siRNA targeting Smad2 (**c)**, Smad3 (**d**), and Smad4 (**e**) in the presence of 4 ng/ml TGF-β1 for 24 hours. snc-RNA was used as control.

TGF-β1/Smads signaling is demonstrated to participate in the fibrogenic response by activating HSCs^5^,^7^. Therefore, we determined whether this canonical Smad-dependent pathway also had a role in TGF-β1-driven CD147 expression. As shown in Fig. 3a, after treated with TGF-β1, the protein levels of Smad2, Smad3, and Smad4 showed no changes in LX-2 cells. However the phosphorylations of Smad2 (p-Smad2) and Smad3 (p-Smad3) were elevated in a TGF-β1-dose-dependent manner. Nuclear translocation of Smad4 and its direct binding to target gene promoter are necessary for TGF-β1 signaling. To understand the molecular mechanism of TGF-β1 regulation of CD147 expression, we determined whether Smad4 translocated to the nucleus from cytoplasm with immunofluorescence. As shown in Fig. 3b, Smad4 was distributed in the cytoplasm of LX-2 cells. However, it aggregated to nucleus in the presence of TGF-β1. In this process, CD147 expression was up-regulated significantly in TGF-β1-treated cells compared with that in control cells (Fig. 3a). Then specific small interfering RNAs targeting Smad2 (si-Smad2), Smad3 (si-Smad3), and Smad4 (si-Smad4) were synthesized to validate the Smad proteins regulating CD147 expression. When LX-2 cells were treated with si-Smad2, si-Smad3, and si-Smad4 respectively, the corresponding Smad expressions were silenced, coupled with the fact that the TGF-β1-upregulated CD147 was depressed (Fig. 3c–e). Collectively, these findings suggested that TGF-β1 induced expression of CD147 in HSCs through activation of Smad signaling by the cooperation of Smad2, Smad3, and Smad4.

### Specific binding of Smad4 to the CD147 promoter

**Figure 4:**
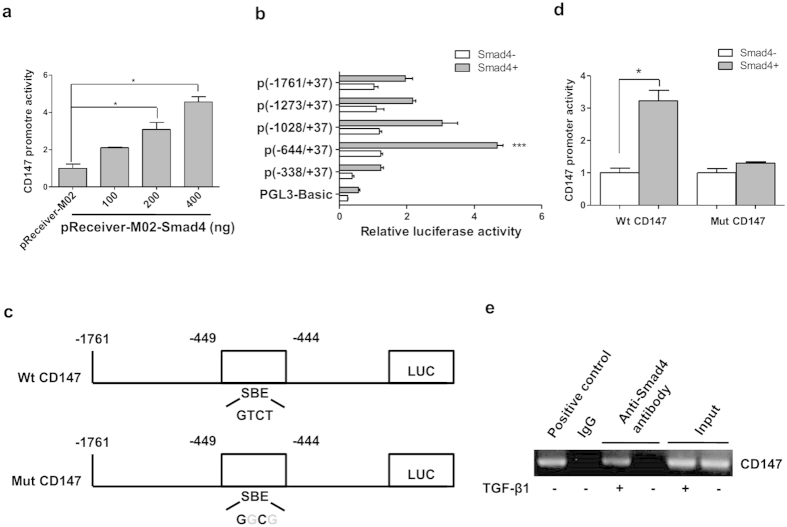
Specific binding of Smad4 to CD147 promoter. (**a**) LX-2 cells were co-tranfected with pGL3-Basic vector containing human CD147 promoter (−1761/+37) and pReceiver-M02-Smad4, CD147 promoter activity was detected by dual-luciferase reporter assay. (**b**) LX-2 cells were co-transfected with different length of CD147 promoters and pReceiver-M02-Smad4, CD147 promoter activity was detected by dual-luciferase reporter assay. (**c**) Schematic representation of wild type (Wt) and mutant (Mut) CD147 promoter regions. (**d**) LX-2 cells were co-tranfected with Wt CD147 or Mut CD147 promoters and pReceiver-M02-Smad4, CD147 promoter activity was detected by dual-luciferase reporter assay. (**e**) LX-2 cells were treated with TGF-β1 and subjected to ChIP assay. Positive control was anti-RNA polymerase. Two percent of the lysate was used as the input control. *P < 0.05.

Previous reports have revealed an indispensable role of Smad4 in TGF-β1-induced expression of a subset of target genes, and it can directly bind to an 8-bp palindromic sequence in the promoter which is called Smad-binding element (SBE)^13^. We thus investigated the transcriptional regulation of Smad4 on CD147 expression using a dual-luciferase reporter assay system. As shown in Fig. 4a, co-transfection of pReceiver-M02-Smad4 and pGL3-Basic vector containing Luc gene under the control of human CD147 promoter (−1761/+37) fragment into LX-2 cells led to a Smad4 dose-dependent increase in luciferase activity. This result indicates that Smad4 has an inducible effect on the activity of the CD147 promoter, which directly regulates the transcriptional expression of CD147.

Next, we developed a series of deletion constructs to identify the minimal promoter required for CD147 activation. We found that the most critical region for transcriptional activity of the CD147 promoter was located in positions between −338 and −644 which involved SBE sequence (Fig. 4b). To further identify the CD147 promoter core region, we then generated a Mut CD147 construct that contained mutations in the SBE domain by site-directed mutagenesis (Fig. 4c). We found that the promoter activity was completely abolished upon mutation of the Smad4-binding site in SBE, suggesting that SBE is required for activation of the CD147 promoter by Smad4 (Fig. 4d). To show direct binding of Smad4 to the putative binding site within the critical CD147 promoter region, chromatin immunoprecipitation assay (ChIP) was performed in LX-2 cells. The PCR-amplified fragment of the CD147 promoter region containing the SBE was retrieved from the immunoprecipitates using an anti-Smad4 antibody, whereas the control fragment could not be (Fig. 4e). These results showed that Smad4 activated CD147 transcription by direct interaction with the SBE domain that was located in the proximal promoter of the CD147 gene.

### ERK1/2 and downstream Sp1 mediated CD147-induced TGF-β1, collagen I, and α-SMA expressions

**Figure 5:**
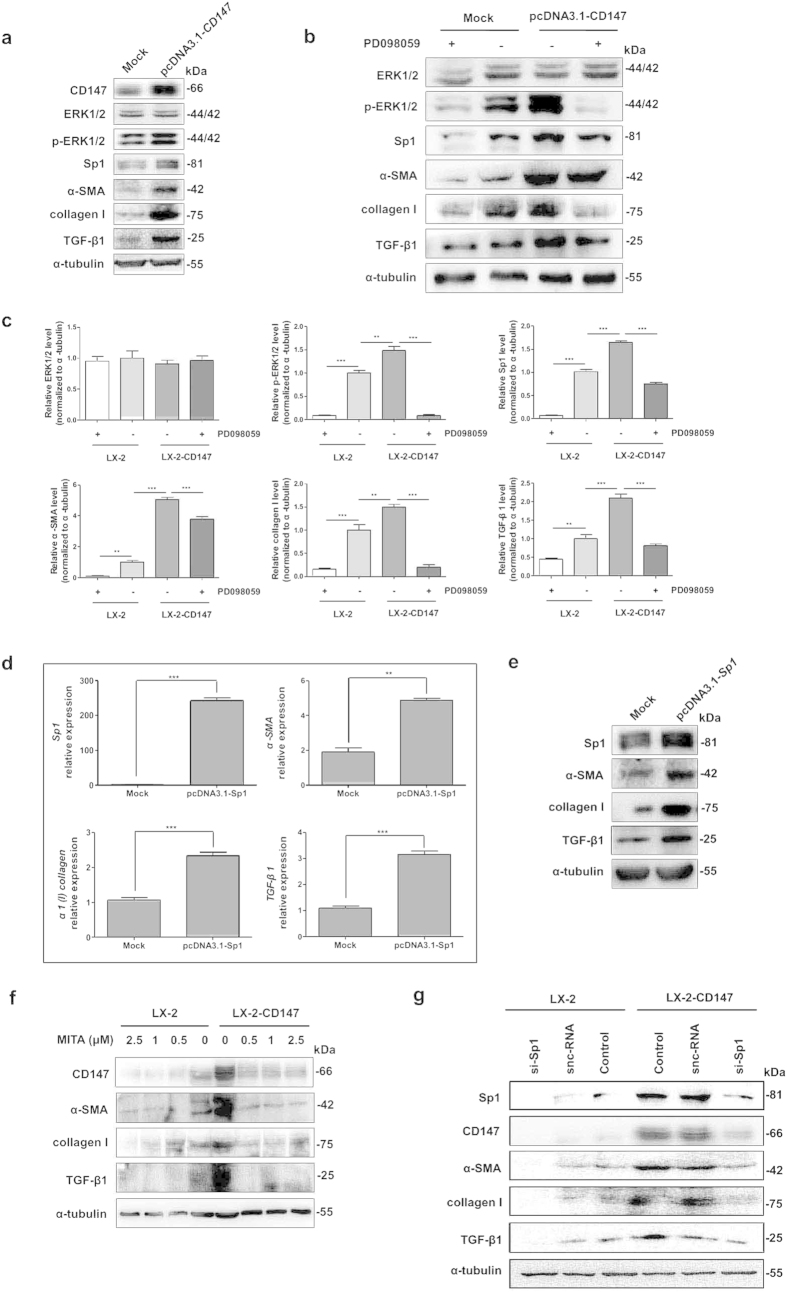
ERK1/2 and Sp1 mediated CD147-induced TGF-β1 and collagen I expressions. (**a**) Western blot analysis of CD147, ERK1/2, p-ERK1/2, Sp1, α-SMA, collagen I, and TGF-β1 in LX-2 cells transfected with pcDNA3.1-CD147, and (**b**) pre-incubated with 1 nM PD098059. (**c**) Western blot scanning densitometry for three independent experiments was analyzed with ImageJ software. Blots were probed for α-tubulin to ensure equal protein loading. (**d**) Real-time RT-PCR and (**e**) western blot analysis of molecules in LX-2 cells transfected with pcDNA3.1-Sp1. Cells were transfected with pcDNA3.1(+) as mock control. (**f**) Western blot analysis of molecules in LX-2-CD147 and LX-2 cells pre-incubated with mithramycin A or (**g**) treated with siRNA targeting Sp1 (si-Sp1). snc-RNA was used as a control. **P < 0.01, ***P < 0.001.

The transient introduction of CD147 strongly induced profibrotic factors such as α-SMA, α1(I) collagen, and TGF-β1 expressions as shown in Fig. 5a. Meanwhile, the levels of phospho-ERK1/2 (p-ERK1/2) and Sp1 in LX-2-CD147 cells were increased compared with control (Fig. 5a). The CD147-upregulated profibrotic factors and Sp1 could be significantly inhibited by the selective ERK1/2 inhibitor PD098059 (Sigma) (Fig. 5b,c). All of these results indicated the crucial involvement of activated MAPK signaling in CD147-dependent TGF-β1 and collagen I production. To clarify the role of Sp1 in CD147-ERK1/2-dependent TGF-β1 and collagen I expression in HSCs, the Sp1 gene was transiently transfected into LX-2 cells followed by detection of fibrosis-related factors. As shown in Fig. 5d,e, over-expression of Sp1 transcription factor significantly induced the α-SMA, α1(I) collagen, and TGF-β1 mRNA and protein up-regulation.

We next wondered whether the feedback effect of CD147 on TGF-β1 expression through Sp1. The treatment of mithramycin A (MITA, from Sigma), a drug known to modify GC-rich regions of the DNA and inhibit Sp1 binding down-regulated the expressions of CD147, α-SMA, α1(I) collagen, and TGF-β1 in both LX-2 and LX-2-CD147 cell lines (Fig. 5f). The RNA interference to knockdown Sp1 expression repressed both basal and CD147-induced α-SMA, α1(I) collagen, and TGF-β1 levels (Fig. 5g). These results suggested that ERK1/2 and downstream Sp1 mediated CD147-induced α-SMA, α1(I) collagen, and TGF-β1 expressions.

### TGF-β1-CD147 positive feedback loop was activated in liver fibrosis

**Figure 6:**
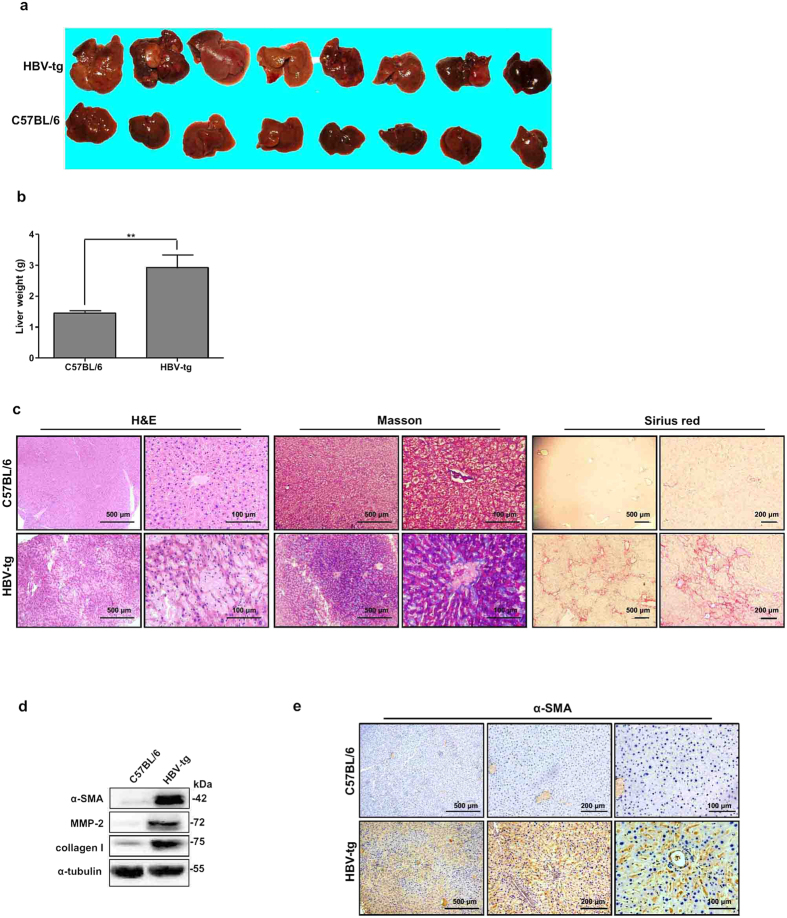
Spontaneous liver fibrosis in HBV-tg mice. (**a**) Morphology of liver and (**b**) liver weight of 12-month-old C57BL/6 and HBV-tg mice (n = 8). **P < 0.01. (**c**) H&E, Masson′s trichrome, and Sirius red stainings of liver tissues from 6-month-old C57BL/6 and HBV-tg mice. (**d**) Western blot analysis of α-SMA, MMP-2, and collagen I expressions in liver tissues from 6-month-old C57BL/6 and HBV-tg mice. (**e**) Imm-unohistochemistry detected α-SMA expression in liver tissues from 6-month-old C57BL/6 and HBV-tg mice.

Spontaneous liver fibrosis is previously reported in HBV transgenic (HBV-tg) mice C57BL/6J-Tg(Alb1HBV)44Bri/J^14^. We used this strain of HBV-tg mice for assessing CD147-TGF-β1 positive feedback loop. It was found that the livers from 12-month-old HBV-tg mice appeared irregular shape and noticeable regenerative nodules with significantly higher weight compared to C57BL/6 control mice (Fig. 6a,b). H&E staining of liver tissues from 6-month-old C57BL/6 mice revealed normal cellular architecture, whereas liver tissues from HBV-tg mice demonstrated intense polymorphonuclear leukocyte and macrophage infiltration with severe centrilobular necrosis (Fig. 6c). Masson′s trichrome staining of the liver was performed to assess the collagen distribution and showed that extensive collagen deposition and pseudolobular formation was evident in liver tissues from HBV-tg mice compared with normal control, which was confirmed by Sirius red staining (Fig. 6c). Accordingly, western blotting and/or immunohistochemistry showed that α-SMA, MMP-2, and collagen I were remarkably increased in livers from HBV-tg mice (Fig. 6d,e). HBx was uniquely expressed (Supplemental Fig. 2a,b). These results verified that liver fibrosis was spontaneously developed in HBV-tg mice.

**Figure 7:**
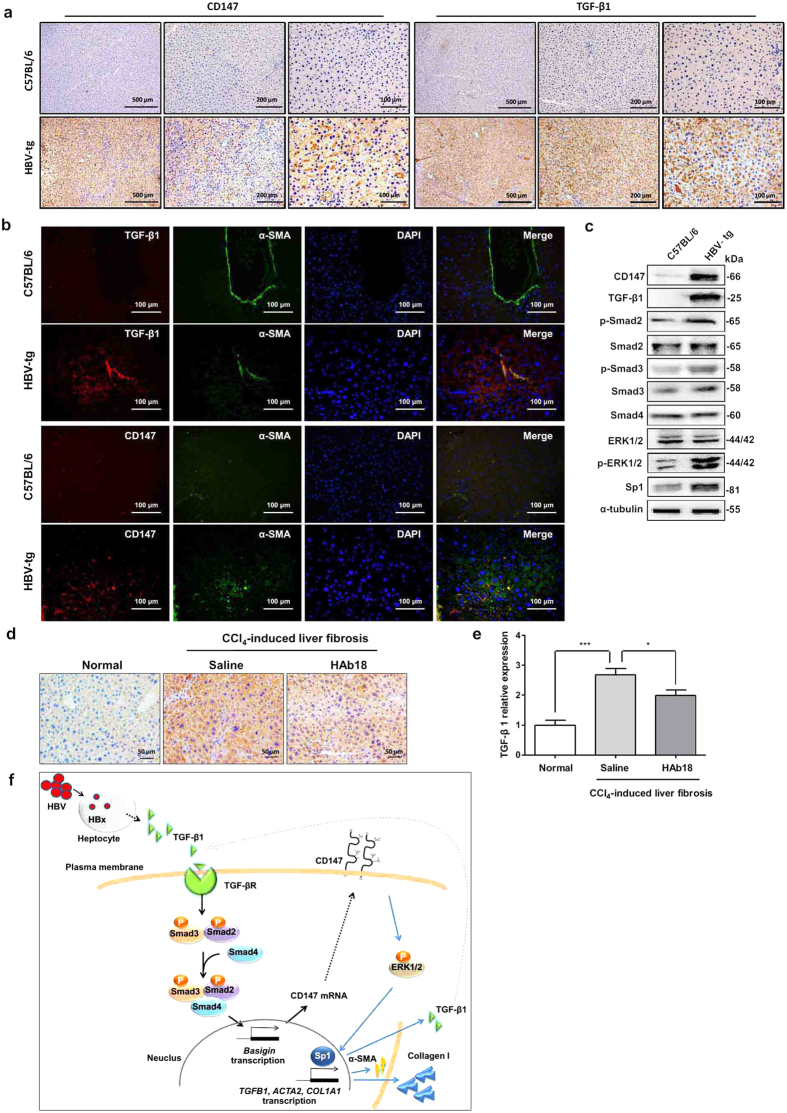
Up-regulation of key molecules of TGF-β1-CD147 positive feedback loop in liver fibrosis mice. (**a**) Immunohistochemistry analysis of CD147 and TGF-β1 expressions in liver tissues from 6-month-old C57BL/6 and HBV-tg mice. (**b**) Double immunofluorescence of TGF-β1 (red), CD147 (red), and α-SMA (green) in liver tissues from 6-month-old C57BL/6 and HBV-tg mice. Cell nuclei were stained with DAPI. (**c**) Western blot analysis of CD147, TGF-β1, p-Smad2, Smad2, p-Smad3, Smad3, Smad4, ERK1/2, p-ERK1/2, and Sp1 in liver tissues from 6-month-old C57BL/6 and HBV-tg mice. (**d**) Immunohistochemistry and (**e**) real-time RT-PCR analysis of TGF-β1 expression in liver tissues from CCl_4_-induced mice after treatment with CD147-specific antibody HAb18. n = 7. (**f**) Schematic model of TGF-β1-CD147 positive feedback loop in HSC activation.

We then checked the expression of key molecules of TGF-β1-CD147 positive feedback loop in liver tissues. Immunohistochemistry analysis displayed a stronger staining of CD147 and TGF-β1 in liver tissues from 6-month-old HBV-tg mice, whereas rare staining of both molecules was observed in control mice (Fig. 7a). Double immunofluorescence detected that both TGF-β1 and CD147 co-localized with α-SMA-positive cells in fibrotic liver tissues, respectively, which suggested that TGF-β1-CD147 signaling in activated HSCs was involved in HBV-related liver fibrosis (Fig. 7b). The activation of all the key molecules (*i.e.* CD147, TGF-β1, p-Smad2, p-Smad3, p-ERK1/2, and Sp1) involved in the regulation of TGF-β1-CD147 positive feedback loop were elevated (Fig. 7c). In our previous work, we demonstrate that liver fibrosis induced by carbon tetrachloride (CCl_4_) is reversed by a monoclonal antibody (HAb18) against CD147^9^. A recent work by other group also shows the attenuation of liver fibrosis progression by blocking CD147 with a specific antibody^15^. To prove the positive feedback from CD147 to TGF-β1, TGF-β1 expression was then detected in CCl_4_-induced mouse liver tissues after treatment of HAb18 antibody. Immunohistochemistry showed that the increased TGF-β1 expression in CCl_4_-induced mouse liver tissue was suppressed by HAb18 antibody (Fig. 7d). The results were verified by real-time PCR analysis that TGF-β1 mRNA expression was significantly decreased in HAb18-treated group compared with saline group (Fig. 7e). Finally, a schematic model of the positive feedback loop was depicted in Fig. 7f.

## Discussion

Chronic infection by HBV is a major cause of liver fibrosis and eventually leads to the development of cirrhosis and HCC^3^. Liver injury by HBV infection releases pro-inflammatory factors, which modulates HSC transdifferentiation contributing to the development of liver fibrosis^16^. HBV encoded-protein, HBx has been reported to induce TGF-β secretion in hepatocytes and subsequently to active HSCs by a paracrine way^11^. We previously find that over-expression of a transmembrane protein CD147 in HSC promotes up-regulation of fibrosis-related genes, including α-SMA, collagen I, and TGF-β1. CD147 is highly expressed in human HBV-related liver cirrhotic tissues and correlated with the Child-Pugh grade. TGF-β1 can also upregulate CD147 expression^9^. Based on the initial studies, it is rationale to assume HBx plays an important role by activation of HSCs through paracrine TGF-β1-CD147 signaling loop in HBV-related liver fibrosis.

TGF-β1/Smads signaling pathway has been reported to play a crucial role in liver fibrosis through induced deposition of ECM, especially type I collagen and secretion of fibrogenic cytokines in hepatocytes and HSCs^5^. However, the specific mechanisms for TGF-β1/Smads stimulating transcription of type I collagen gene in HSC are much unknown. Although the DNA-binding activity of Smad3 and Smad4 is weak and they usually associate with other DNA-binding transcription factors for target gene transcription, Smad4 directly participating the transcriptional regulation is disclosed in epithelial cells^8^,^17^. In the study, we found that the presence of Smad2, Smad3, and Smad4 was necessary for CD147 expression, and a Smad4-binding DNA element was located in the human basigin gene promoter for CD147 transcription in HSC cells.

Earlier studies show Sp1/3 independent or cooperative with Smad3/4 or Smad2/4 for α2(I) collagen (COL1A2) and α1(I) collagen (COL1A1) transcription^18^,^19^,^20^. Sp1 and AP-1 form a protein-DNA transcriptional regulatory complex for transcriptionally activating TGF-β1 (gene TGFB1) promoter in hepatitis C virus-infected hepatoma cells^21^. It is reported that the transcriptional activity of Sp1 in TGF-β1 regulation can be repressed with transcription factor Nrf2 by interacting with c-Jun^22^. Sp1/3 is also required for α-SMA (gene ACTA2) enhancer activation in response to TGF-β1 stimulation myofibroblast maturation^23^. We here demonstrated that the CD147 up-regulation by TGF-β1/Smad4 mediated Sp1 in induction of α1(I) collagen, TGF-β1, and α-SMA in human HSCs. As an upstream factor, ERK1/2 can modulate Sp1 activation and mediates TGF-β1 regulation for target genes^24^. As TGF-β1 can also up-regulate and modulate Sp1 expression and activation^25^ which was also proved in our study, we here showed CD147 as an upstream molecule to induce ERK1/2 phosphorylation, which resolved the signaling context between TGF-β1 and Sp1.

As integrin is important for HSC transdifferentiation and an interaction between CD147 and integrin is shown by our previous study^26^,^27^, CD147 mediated ERK1/2 activation probably by directly binding with integrin in HSCs. Taken together, the activation of TGF-β1-CD147 signaling loop was involved with Smads and ERK/Sp1 transduction to induce α-SMA and type I collagen over-production, which favored activated phenotypes of HSCs, such as enhanced wound closure, cell contraction, and cell proliferation^9^.

HBV transgenic mouse models are widely used for study of HCC development^28^,^29^. A strain of HBV transgenic mouse C57BL/6J-Tg(Alb1HBV)44Bri/J is also observed the development of liver fibrosis^14^,^30^, which was verified in our study. The HBV-tg mice used in this study contain sequence encoding the HBV large envelope polypeptide under the transcriptional control of the mouse albumin promoter. They also contain the HBV enhancer element and the HBV X open reading frame and its putative promoter sequences. Despite the donating investigators report a major decrease in the steady-state level of HBV envelope-specific transcripts and no HBV X-specific transcripts in tumors by Northern blot analysis^31^, we detected a high expression level of HBx by western blot and immunohistochemistry analysis in non-tumoral liver tissues from 6-month-old mice. Currently, a specific marker for the identification of activated HSCs is unavailable. However, α-SMA-positive HSCs can be distinguished from the other portal, interface, and septal myofibroblasts by their specific position in the liver perisinusoidal space^32^. In our previous work^9^, we identify the co-localization of CD147 molecule with α-SMA-positive cells in liver perisinusoidal space both in CCl_4_-induced fibrotic liver and human cirrhotic liver tissues. In our recently published work^33^, a significantly positive correlation is observed between CD147 and α-SMA (*r* = 0.8857, *P* = 0.0333) at N-diethylnitrosamine/phenobarbital initiation and early stage of liver tumor formation. As a mesenchymal phenotype marker, α-SMA is also expressed in transformed hepatocytes during the process of epithelial-mesenchymal transition^34^. Our previous study finds that α-SMA is detected in the cytoplasm of hyperplastic hepatocytes in CCl_4_-induced mouse fibrotic liver sections^35^. In the spontaneous liver fibrosis HBV-tg model, we also observed the positive staining of α-SMA in hepatocytes as well as the cells in perisinusoidal space in liver tissues (Fig. 7b, green fluorescence). The merge of CD147-positive red fluorescence with α-SMA-positive green fluorescence in perisinusoidal space was obviously presented, which indicated that CD147 was over-expressed in activated HSCs in HBV-tg mouse liver.

In summary, we here demonstrate that TGF-β1 released from HBx-expressing hepatocytes promotes the activation of HSCs through up-regulating CD147 by TGF-β1/Smads signaling in the development of liver fibrosis. As a bridge molecule, CD147 links TGF-β1 and Sp1 signaling transduction for transcription of collagen I, α-SMA, and TGF-β1 itself, therefore a positive feedback between TGF-β1 and CD147 is proposed in HSC transdifferentiation. Our findings provide new insights into HBV-mediated fibrogenesis. A new strategy using combination of TGF-β receptor inhibitor and anti-CD147 antibody may be promised to reverse the liver fibrosis in future.

## Materials and Methods

### Cell culture

Human HSC cell line, LX-2 and human normal liver cell line, L02 were cultured at 37 °C in a humidified atmosphere containing 5% CO_2_ with Dulbecco′s Modified Eagle Medium (DMEM) (Hycolon, Logan, USA) containing 10% fetal bovine serum, 100 U/ml of penicillin, and 100 mg/ml of streptomycin. The L02-HBx cell line was established by stable transfection of eukaryotic expression plasmid pcDNA3.1-HBx with hygromycin B selection. LX-2-CD147 cell line was established by infecting lentivirus-mediated human CD147 cDNA into LX-2 cells with puromycin selection for stable transfection.

### Western blot

Western blot was performed as previously described^36^. The primary antibodies used were mouse-anti human CD147 antibody (1:1000) prepared by our laboratory^37^, rat anti-mouse CD147 (1:500), anti-HBx (1:1000), anti-α-SMA (1:1000), anti-α1(I) collagen (1:1000) (Abcam, Cambridge, UK), anti-Smad2 (1:500), anti-phospho-Smad2 (1:500), anti-ERK1/2 (1:500), anti-phospho-ERK1/2 (1:500) (Cell Signaling Technology, Danvers, USA), anti-MMP-2 (1:500), anti-Sp1 (1:500), anti-Smad3 (1:500), anti-phospho-Smad3 (1:500) (Epitomic, California, USA), anti-Smad4 (1:1000) (Santa Cruz Biotechnology, Santa Cruz, USA), anti-TGF-β1 (1:200), and anti-α-tubulin (1:200) (Santa Cruz Biotechnology). Secondary antibodies included horseradish peroxidase-conjugated goat anti-mouse IgG(H+L) (1:3000), goat anti-rat IgG(H+L) (1:2000) and sheep anti-rabbit IgG(H+L) (1:2000) (Pierce Biotechnology, Rockford, USA). The Western-Light chemiluminescent detection system (Image Station 4000 MM Pro, XLS180, Kodak, USA) was used to visualize the signals. The gray values were analyzed with ImageJ software.

### Enzyme-linked immunosorbent assay (ELISA)

L02 and L02-HBx cells were plated in 6-well cell culture plates at a density of 1 × 10^6^ cells per well for 24 h. Cells were washed and starved for 24 h with serum-free DMEM. Then conditioned medium was harvested and centrifuged at 3000 g for 5 min to remove cell debris and stored at −20 °C until use. Soluble TGF-β1 was performed using a TGF-β1 Emax® Immune Assay System ELISA kit (Peprotech, Madison, WI) according to the manufacturer′s instructions. The concentration in each sample well was determined by interpolation from a standard curve. Each sample was tested in duplicate.

### Real-time RT-PCR

RNA was extracted with the Total RNA Kit II (Omega, Riverside, USA) and reverse transcribed into cDNA using a PrimeScript™ RT reagent kit (TaKaRaBio, Otsu, Japan). Single-stranded cDNA was amplified by quantitative RT-PCR using a SYBR Premix ExTaq™ kit (TaKaRaBio) on the Stratagene Mx3005P™ Real-Time PCR System (Agilent Technologies, Germany). Glyceraldehyde-3-phosphate dehydrogenase mRNA was used to normalize RNA inputs. RT reactions were performed at 42 °C for 60 min followed by 70 °C for 10 min. The qRT-PCR was conducted at 95 °C for 20 sec, followed by 40 cycles of 95 °C for 10 sec, 60 °C for 20 sec, and then 70 °C for 10 sec. The occurrence of primer dimers and secondary products was inspected using melting curve analysis. Our data indicated that the amplification was specific. There was only one PCR product amplified for individual set of primers. All primers were synthesized by Shanghai Sangon Biological Engineering Technology & Services Co., Ltd. and listed in Supplemental Table 1.

### Wound healing assay

LX-2 cells were grown to 100% confluence on 18-mm-diameter dishes. The growth medium was removed, cells were washed and a wound was produced (0.5 mm). Cells were rinsed to remove non-adherent cells, and fresh medium with 1% fetal bovine serum was added. Digital images were taken at time points with an inverted microscope (CKX41; Olympus) equipped with digital camera.

### Transwell migration assay

LX-2 and LX-2-CD147 cells were harvested and re-suspended to give a final concentration of 1.0 × 10^6^/ml in serum-free DMEM. Cell suspensions (100 μl) were added to the upper compartment, and the filter chamber was inserted in a 24-well plate with DMEM containing 10% fetal bovine serum. The plate was incubated for 12 h at 37 °C in a 5% CO_2_ atmosphere. Then the filters were fixed with methanol for 10 min and stained with 0.5% methylrosaniline chloride for 20 min. The cells on the upper surface of the filters were removed with a cotton swab. The cells on the reverse side were counted under a microscope in five random fields at a magnification of ×100. Each assay was performed in triplicate.

### Cell contraction assay

A standard kit assay was used (Cell Biolabs, San Diego, CA, USA). LX-2 cells were harvested and re-suspended in DMEM, two parts of cells were mixed with eight parts of collagen gel lattice mixture and plated for 1 h at 37 °C. After the gel was polymerized, 1 ml of medium was added and incubated for 2 days. Next the gels were released from the sides of wells, and 24 h later the images were taken. The changes of collagen gel size were analyzed using ImageJ software and normalized to areas of the well.

### RNA Interference

Transfection of small interfering RNAs (siRNA) was performed using Lipofectamine 2000 (Invitrogen). All siRNA sequences were synthesized by Shanghai GenePharma Co, Ltd. and were listed in Supplemental Table 2. The snc-RNA was used as a negative control under similar conditions.

### Immunofluorescence

Immunofluorescence was carried out as described previously^36^. Primary antibodies included rabbit anti-Smad4 (Santa Cruz Biotechnology), mouse anti-α-SMA (Abcam), rabbit anti-TGF-β1 (Biorbyt, Cambridge, UK), and rat anti-mouse CD147 antibodies (Abcam). Alexa Fluor 488-conjugated donkey anti-rabbit IgG(H+L) (Molecular probes), Alexa Fluor 488-conjugated goat anti-mouse IgG(H+L) Alexa Fluor 594-conjugated donkey anti-rabbit IgG(H+L), and Alexa Fluor 594-conjugate donkey anti-rat IgG(H+L) (Pierce, Rockford, USA) were used as secondary antibodies. The 4′,6-diamidino-2-phenylindole (DAPI) was used as a nuclear counterstain (Molecular probes, Eugene, USA). The co-localization expression was visualized under a confocal fluorescence microscopy (Nikon, Japan).

### Immunohistochemistry

Liver tissues were fixed with 4% formalin and embedded in paraffin. Sections were deparaffinized and incubated with primary antibodies including anti-HBx, anti-α-SMA, anti-CD147 (Abcam), and anti-TGF-β1 (Biorbyt), followed by visualization with the Histostain®-Plus Kit (Invitrogen).

### Collagen staining

Collagen accumulation was detected with Sirius red and Masson’s trichrome, which were performed as previously described^38^. Liver tissues were fixed with 4% formalin and embedded in paraffin. Sections were cut at a 5 μm thickness. Following deparaffinization and hydration, the sections were stained with Sirius red and Masson′s trichrome (Sigma, USA).

### Dual-luciferase reporter assay

The 1659-base pair cDNA containing the coding region of human Smad4 (pReceiver-M02-Smad4) was purchased from GeneCopoeia TM (Rockville, MD, USA). A reporter construct plasmid P(−1761/+37), which contained a 1798-bp genomic DNA fragment spanning the 5′ upstream region of CD147, and a series of deletion constructs were generated by our laboratory^39^. To generate site-directed mutants of CD147 binding elements at −449 to −444, the QuikChange® Site-Directed Mutagenesis Kit (Stratagene, La Jolla, CA, USA) was used. The incorporation of the mutations was verified by sequencing (Shanghai Sangon). The CD147 promoter plasmids containing the firefly luciferase reporter were co-transfected with pReceiver-M02-Smad4 and internal control, pRL-TK (Promega, Madison, WI, USA), using Lipofectamine 2000 (Invitrogen). Twenty-four hours after transfection, cells were detected for luciferase activity using the Dual-Luciferase Reporter Assay System (Promega).

### Chromatin immunoprecipitation (ChIP)

This was done essentially as described previously^36^. LX-2 cells were treated with 4 ng/ml of TGF-β1 for 24 h. ChIP was performed using the EZ ChIP™ Chromatin Immunoprecipitation Kit (Millipore Bedford, MA, USA). Cell lysates were incubated with anti-Smad4 (R&D Systems, Minneapolis, USA) or IgG antibodies. The immunoprecipitated DNA was amplified by the promoter-specific primers: forward 5′-AGGTCACTTCCTCCCACC-3′; reverse 5′-TCTGAGTTAAACACGGGC-3′. The PCR products were analyzed on 1% agarose gel.

### Mice

HBV transgenic mice C57BL/6J-Tg(Alb1HBV)44Bri/J, which contains HBV genome S, pre-S, and X domains under the mouse albumin promoter, were provided by The Jackson Laboratory (Bar Harbor, ME). C57BL/6 mice were purchased from VITALRIVER experiment animal company (Beijing, China). The establishment and evaluation of liver fibrosis mouse model and treatment with antibody against CD147 were reported in our previous study^9^. All experimental protocols were approved by Laboratory Animal Ethics Committee of Fourth Military Medical University and performed in strict accordance with the People′s Republic of China Legislation Regarding the Use and Care of Laboratory Animals.

### Statistical analysis

Each experiment was repeated at least three times. Student′s t-test was used to compare the two mean values. A one-way analysis of variance was performed to compare the multiple mean values. Data were presented as the mean ± SD from three independent experiments unless otherwise indicated. The Graphpad Prism software and SPSS 17.0 software were used for statistical analysis.

## Additional Information

**How to cite this article**: Li, H.-Y. *et al.* Activation of TGF-β1-CD147 positive feedback loop in hepatic stellate cells promotes liver fibrosis. *Sci. Rep.* **5**, 16552; doi: 10.1038/srep16552 (2015).

## Supplementary Material

Supplementary Information
