# Supplementary material for: Activation of TGF-β1-CD147 positive feedback loop in hepatic stellate cells promotes liver fibrosis
Source: Sci Rep. 2015 Nov 12;5:16552. doi: 10.1038/srep16552 (PMC4642271; doi:10.1038/srep16552)
Supplement: Supplementary Information [file srep16552-s1.doc]

Supplementary information for

**Activation of TGF-β1-CD147 positive feedback loop in hepatic stellate cells promotes liver fibrosis**

Hai-Yan Li^1,2*^, Di Ju^1*^, Da-Wei Zhang^1,3^, Hao Li^1^, Ling-Min Kong^1^, Yanhai Guo^4^, Can Li^1^, Xi-Long Wang^1^, Zhi-Nan Chen^1^, Huijie Bian^1^

^1^ Cell Engineering Research Center and Department of Cell Biology, State Key Laboratory of Cancer Biology, Fourth Military Medical University, Xi′an 710032, China

^2^ Department of Medical Technology, Xi′an Medical University, Xi′an, 710021 China

^3^ Research Center for Biological Therapy, Beijing 302 Hospital, Beijing, China

^4^Department of Pharmacogenomics, School of Pharmacy, Fourth of Military Medical University, Xi′an, 710021 China

^*^ Hai-Yan Li and Di Ju contributed equally to this work.

**Correspondence:**

Huijie Bian Prof., PhD (E-mail: hjbian@fmmu.edu.cn)

Zhi-Nan Chen Prof., PhD (E-mail: znchen@fmmu.edu.cn)

This PDF file includes:

Supplementary Figure 1 to 2

Supplementary Table 1 to 2

**Supplementary Figures and Tables:**


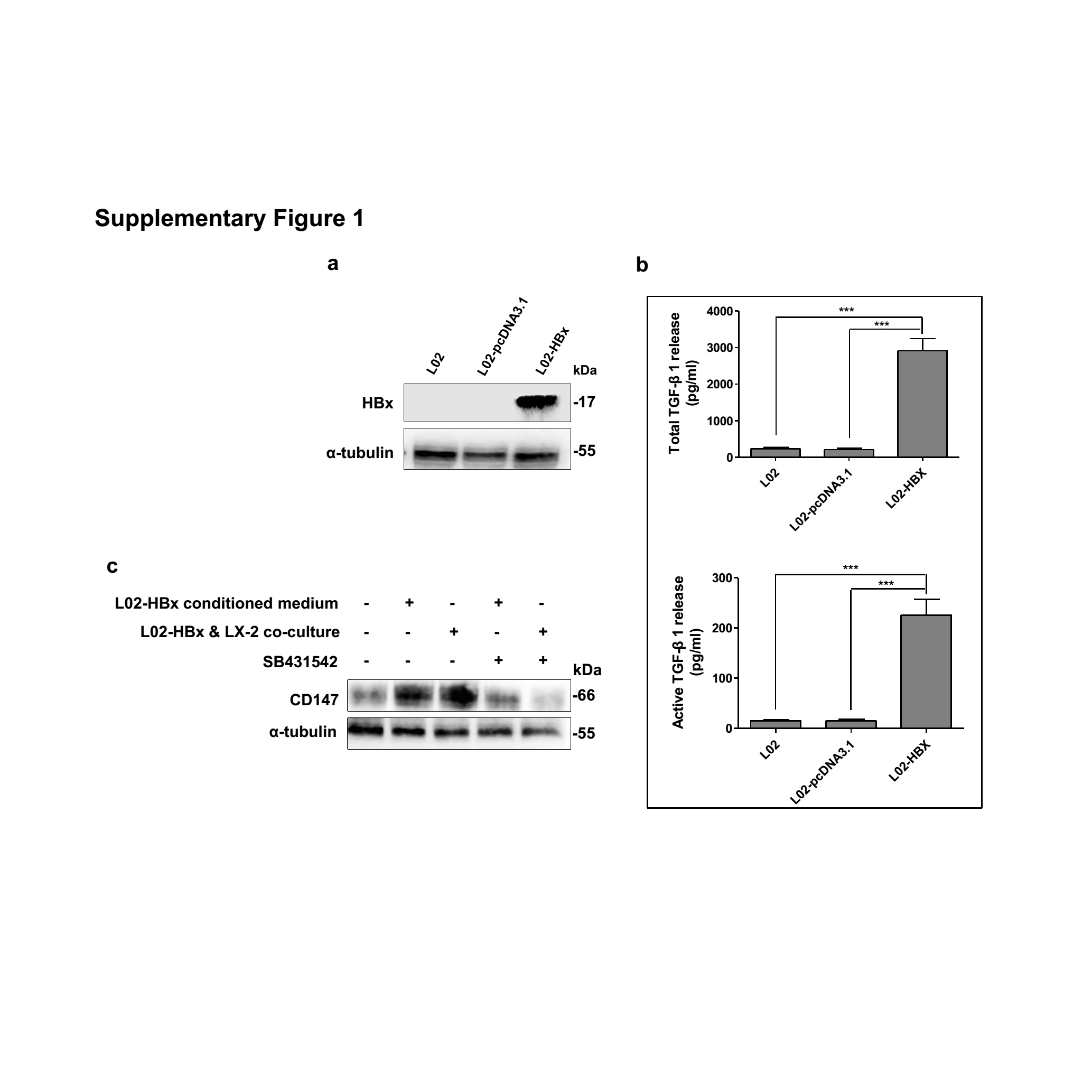


**Supplementary Figure 1. HBx regulated CD147 expression in HSCs through paracrine TGF-β1.** (a) Western blot analysis of HBx expression in HBx-stably transfected non-tumor hepatic L02 cells. Non-transfected L02 and mock-transfected L02-pcDNA3.1 cells were as controls. (b) ELISA detection of total and active forms of TGF-β1 in L02-HBx. ****P* < 0.001. (c) Western blot analysis of CD147 expression in LX-2 cells treated with conditioned medium from L02-HBx cells and co-cultured with L02-HBx cells in a non-contact system in the presence of SB431542.


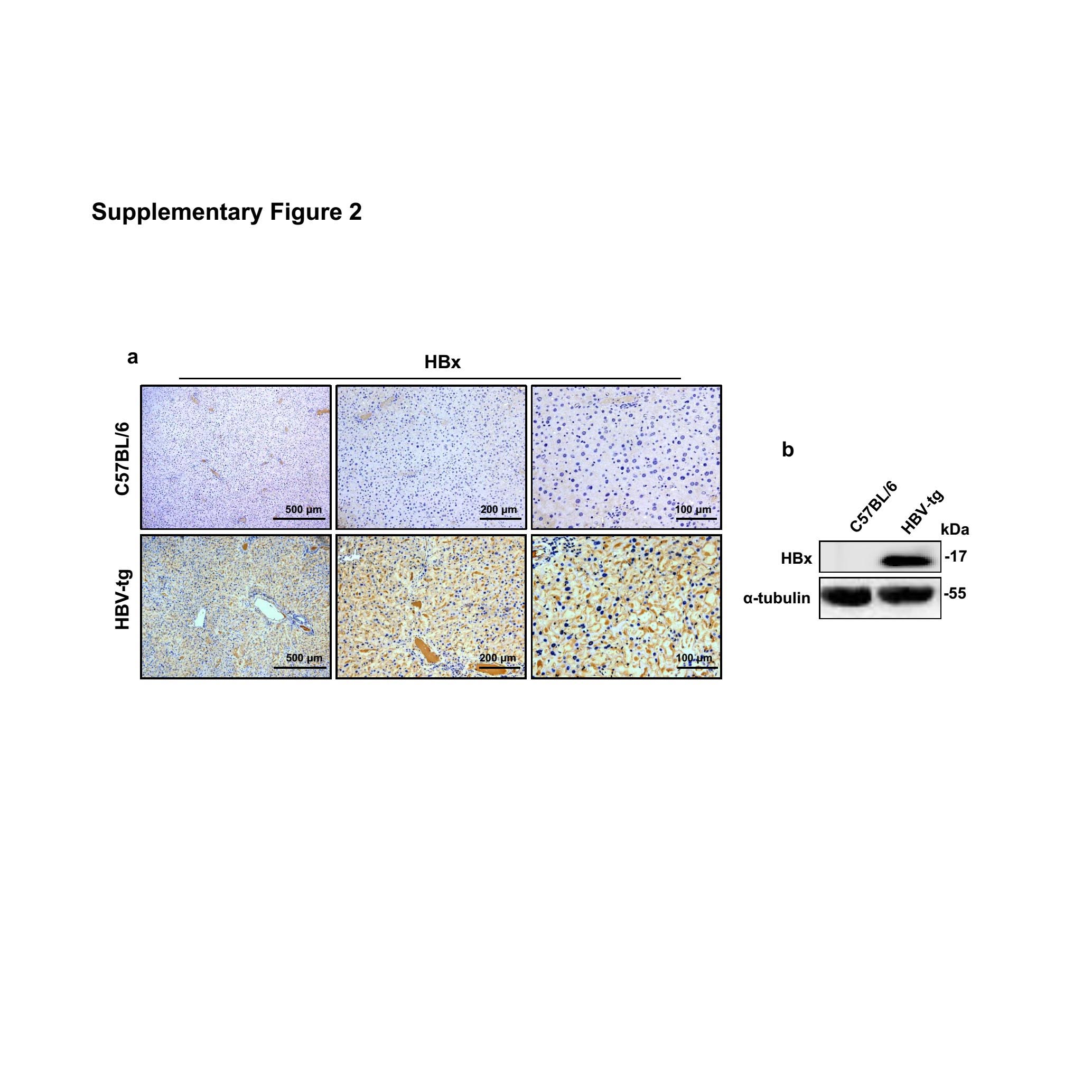


**Supplementary Figure 2. High expression of HBx was detected in HBV-tg mice.** (a) Immunohistochemistry detected HBx expression in liver tissues from 6-month-old C57BL/6 and HBV-tg mice. (b) Western blot analysis of HBx expression in liver tissues from 6-month-old C57BL/6 and HBV-tg mice.

**Supplementary Table 1 Sequences of PCR primers**

| Gene | Forward sequence (5′-3′) | Reverse sequence (5′-3′) |
| --- | --- | --- |
| Human CD147 | ACTCCTCACCTGCTCCTTGA | GCCTCCATGTTCAGGTTCTC |
| Human TGF-β1 | GGCAGTGGTTGAGCCGTGGA | TGTTGGACAGCTGCTCCACCT |
| Human α-SMA | GGCTCTGGGCTCTGTAAGG | CTCTTGCTCTGGGCTTCATC |
| Humanα1(I) collagen | AACATGACCAAAAACCAAAA | CATTGTTTCCTGTGTCTTCTGGGTG |
| Human MMP-2 | GGCAGTGCAATACCTGAACACC | GTCTGGGGCAGTCCAAAGAACT |
| Human Sp1 | AATTTGCCTGCCCTGAGTGC | TTGGACCCATGCTACCTTGC |
| Human GAPDH | GCACCGTCAAGGCTGAGAAC | TGGTGAAGACGCCAGTGGA |
| Mouse TGF-β1 | TGCGCTTGCAGAGATTAAAA | CTGCCGTACAACTCCAGTGA |
| Mouse GAPDH | AGGTCGGTGTGAACGGATTTG | TGTAGACCATGTAGTTGAGGTCA |

**Supplementary Table 2 Sequences of si**RNAs

| siRNA | Sense sequence (5′-3′) | Antisense (5′-3′) |
| --- | --- | --- |
| si-CD147  si-Smad2 | GAGGAACUCACGAAGAACTTGGUGUUCGAUAGCAUAUUATT | GUUCUUCGUGAGUUCCUCTTUAAUAUGCUAUCGAACACCTT |
| si-Smad3 | GCAACCUGAAGAUCUUCAATT | UUGAAGAUCUUCAGGUUGCTT |
| si-Smad4 | GGUGGAGAGAGUGAAACAUTT | AUGUUUCACUCUCUCCACCTT |
| si-Sp1 | CCUGGAGUGAUGCCUAAUATT | UAUUAGGCAUCACUCCAGGTT |
